# Supplementary material for: Association between oral microbiome and seven types of cancers in East Asian population: a two-sample Mendelian randomization analysis
Source: Front Mol Biosci. 2023 Nov 21;10:1327893. doi: 10.3389/fmolb.2023.1327893 (PMC10702768; doi:10.3389/fmolb.2023.1327893)
Supplement: Supplementary file 1 [file DataSheet1.ZIP › Supplementary Materials/MR plots for tongue/tongue═╝/Lung cancer/pheno.1659_to_lung cancer_forest.pdf]

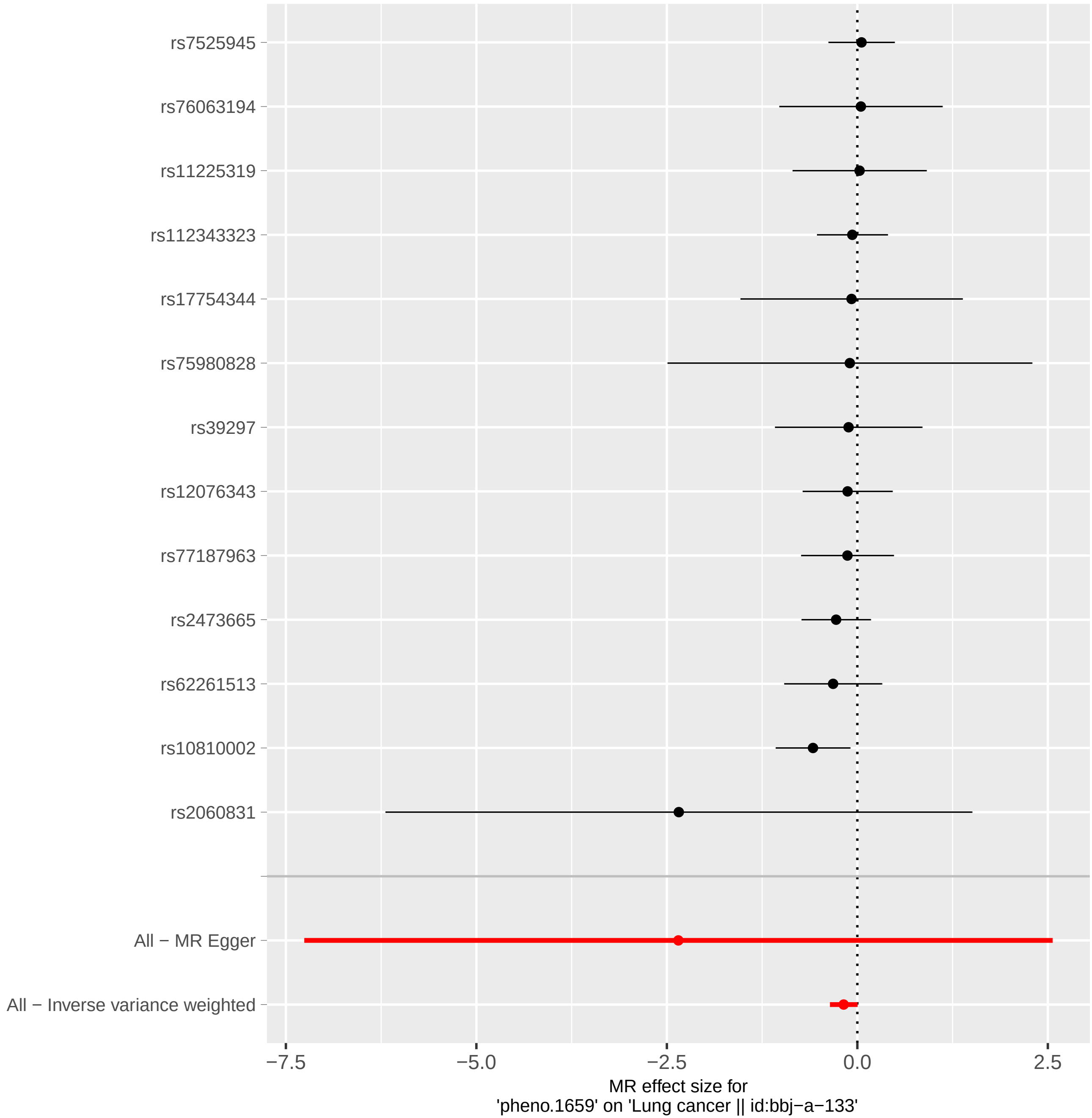

rs7525945
rs76063194
rs11225319
rs112343323
rs17754344
rs75980828
rs39297
rs12076343
rs77187963
rs2473665
rs62261513
rs10810002
rs2060831
All − MR Egger
All − Inverse variance weighted
−7.5
−5.0
−2.5
0.0
2.5
MR effect size for
'pheno.1659' on 'Lung cancer || id:bbj−a−133'
